# A theory-based exploration of antibiotic use in primary healthcare in Gezira state, Sudan

**DOI:** 10.1186/s43058-021-00229-3

**Published:** 2021-12-04

**Authors:** Anna-Leena Lohiniva, Einas Elwali, Duha Abuobaida, Ashwag Abdulrahim, Paul Bukuluki, Maha Talaat

**Affiliations:** 1 WHO Eastern Mediterranean Regional Office, Cairo, Egypt; 2 Federal Ministry of Health, Khartoum, Sudan; 3 National Health Insurance Fund, Khartoum, Sudan; 4 WHO Country Office, Khartoum, Sudan

**Keywords:** Behaviour change, Theory-based intervention, Antibiotic use, Primary healthcare

## Abstract

**Background:**

Inappropriate use of antibiotics is a major contributing factor to the emergence of antimicrobial resistance globally, including in Sudan.

**Objectives:**

The project aimed to develop a theory-driven behaviour change strategy addressing both prescribers and patients based on factors that are driving antibiotic use in primary healthcare settings in Gezira state in Sudan.

**Methods:**

The strategy was designed based on the Theoretical Domains Framework (TDF) to identify behavioural domains and the Behaviour Change Wheel (BCW) to select appropriate intervention functions. The process included (1) a formative qualitative research study and (2) a knowledge co-production workshop that utilized the results of the qualitative study to design a salient, appropriate, and credible behaviour change strategy.

**Results:**

The TDF domains related to prescribers that emerged from the study included knowledge, skills, and intention. The selected BCW intervention functions included education, training, modelling, and persuasion. The main TDF domains related to patients included social influences and intention. The selected BCW intervention functions included enablement and education.

**Conclusion:**

Using the TDF and BCW intervention functions, the study identified behavioural domains that influence antibiotic prescription and consumption in rural primary healthcare settings in Gezira state in Sudan and appropriate intervention functions to modify these behaviours. Knowledge co-production ensured that the evidence-based strategy was acceptable and practical in the local context.

Contributions to literature

- This paper identifies barriers to changing antibiotic use in the primary healthcare setting in Sudan. There is limited knowledge of barriers to changing antibiotic prescription practices globally, including in Sudan.
- Literature points out the importance of understanding behavioural barriers within a given context. This paper provides evidence of context-specific behaviour change barriers that can be utilized to develop a strategic behaviour change intervention in Sudan. The findings can be utilized in other similar socio-cultural settings.
- The impact of antibiotic prescription practices on reducing antimicrobial resistance can be shown when the interventions are implemented on a large scale. The project showed that the TDF and BCW together with knowledge co-creation can be can be used effectively in the context of Sudan to offer insights for a scalable intervention that is practical, acceptable, and implementable.

## Introduction

The use of antibiotics without a clear clinical indication is a major driving force of antimicrobial resistance (AMR) worldwide [1, 2]. It has been estimated that up to 50% of all antimicrobials prescribed to people globally are unnecessary [3]. AMR leads to higher medical costs, prolonged hospital stays, and increased mortality. There is an urgent need to change the way antibiotics are prescribed and consumed to preserve antibacterial therapy, which has drastically reduced deaths and complications caused by bacterial infections and set the stage for modern medicine [3].

The inappropriate prescription of antibiotics is common in Sudan across different types of facilities and in various geographic regions in the country [4–12]. Studies also show that patients in Sudan commonly request antibiotics when visiting health facilities [13]. According to a Sudan pharmaceutical sector assessment in 2018, nationally over 50% of patients visiting primary healthcare centres received antibiotics, the majority of which were broad-spectrum and second generation antibiotics [14]. The national antibiotic treatment guidelines instruct prescribers to prescribe an antibiotic only when indicated and to select a narrow spectrum antibiotic for minor health problems. The majority of patients in primary healthcare settings seek care for upper respiratory tract infections (that are mostly viral and do not require antibiotic treatment), malaria, and urinary tract infections. Severe infections are referred to secondary and tertiary healthcare facilities indicating that most patients visiting primary healthcare settings only have minor health problems that can be treated with narrow spectrum antibiotics [15]. Third generation antibiotics are only available in secondary and tertiary care hospitals, not in rural health units, but they can be purchased in community pharmacies. Culture sensitivity testing in the primary healthcare centres is rare; instead, prescribers make treatment decisions by assessing medical signs and symptoms.

Methods for modifying antimicrobial prescription practices remains unclear and seems to vary significantly from one setting to another [16, 17], which highlights the need to develop context specific-behaviour change approaches. Context-specific behavioural and cultural determinants influence antibiotic prescribing. Understanding them and intervening is paramount to behaviour change [18]. There is also increasing evidence that the use of theory to understand behaviour change mechanisms is necessary to improve the effectiveness of interventions. Behavioural change has been shown to be more effective if interventions are based on principles drawn from evidence and theories of behaviour and behavioural change [19, 20].

The Ministry of Health (MOH) in Sudan, in collaboration with the WHO, implemented a theory-based behaviour change project entitled “Tailoring Antimicrobial Resistance (AMR) Program (TAP)”. The TAP project is linked with the WHO global action plan for AMR that calls for countries to raise awareness and modify antibiotic use related behaviours [21], and it is linked with the Sudan national action plan for AMR that was endorsed in 2018 [22]. The TAP project plan included piloting a behaviour change intervention to better align antibiotic prescription practices with the national antibiotic prescription guidelines in the rural healthcare setting, which provides 60% of health services in Sudan [15]. Of particular interest in this regard was the use of broad spectrum antibiotics as the national guidelines recommend the use of narrow spectrum antibiotics to treat mild health problems as well as the use of antibiotics for mild viral upper respiratory infections, which is not recommended in the national guidelines [15]. During the initial TAP project phase, prescribers from the primary healthcare units and other project stakeholders from various levels of the health system raised concerns about patient behaviours, most notably that they ask for antibiotics in the health centres even if they are not recommended by the medical doctors. Prescribers believed that the pressure from patients was so high that they were not able to change their current antibiotic prescription practices unless the behaviour of patients was also addressed.

TAP is a systematic methodology based on the Theoretical Domains Framework (TDF) used to identify underlying influences on behaviours for adopting appropriate antibiotic prescription and consumption behaviours. TAP also uses the Behaviour Change Wheel (BCW) to select appropriate intervention functions that feed into a behaviour change strategy. The TDF and BCW were selected as frameworks as they are increasingly used to provide a systematic and theoretical basis for understanding and changing behaviour [20, 23–28] and they were seen as practical enough to be used with a multidisciplinary group of public health experts. The methodology included formative qualitative research based on the TDF framework and utilization of the findings to suggest appropriate behaviour change interventions and activities based on the BCW during a knowledge co-production workshop.

The paper describes barriers and facilitators to reduce the prescription and consumption of antibiotics in the primary healthcare level in Sudan and the development of intervention functions for a behaviour change strategy.

## Methodology

### The setting

The TAP project was conducted in Madani, the capital city of Al Gazira state in the east-central region of Sudan. The city’s population is 3.5 million, 70% of the more than 5 million in the entire state. Al Gazira has 93 hospitals and 984 primary health care centres (PHCC) the majority of which are in Madani [29].

### Design

The TAP behaviour change strategy development process was based on two essential components: (A) a formative qualitative research study that aimed to explore the factors influencing physicians’ antibiotic prescribing practices as well as patient demand for antibiotics and (B) a knowledge co-production workshop that utilized the findings of the qualitative research to design a salient, legitimate, and credible behaviour change strategy pilot [30–35].

### Qualitative research study

#### Theoretical framework

The qualitative study used the theoretical domains framework (TDF) to explore prescriber and patient perspectives on the influences on inappropriate use of antibiotics. The framework consists of 14 domains that include (1) knowledge, (2) skills, (3) social/professional role and identity, (4) beliefs about capabilities, (5) optimism, (6) beliefs about consequences, (7) reinforcement, (8) intentions, (9) goals, (10) memory, attention and decision processes, (11) environmental context and resources, (12) social influences, (13) emotion, and (14) behavioural regulation [25, 26, 28]. The domains were defined and adapted for the context. See TDF domains and definitions in Table 1. The findings of the qualitative analysis that were organized to TDF domains were further matched with the COM-B model, which is based on capability, opportunity, and motivation, all of which influence behaviour. These were further matched with BCW intervention functions: education, persuasion, incentivization, coercion, training, restriction, training, environmental restructuring, and enablement [36].

**Table 1:** Definitions of the domains of Theoretical Domains framework (TDF) as adapted for the TAP

Domain	Definition
Knowledge	Knowledge of antibiotics and antimicrobial resistance
Skills	Communication and negotiation skills to answer patient demand
Memory, attention, and decision process	Cognitive abilities to make decisions to take or prescribe antibiotics
Behaviour regulation	Having made plans to change behaviour
Social influences	Social networks, social norms
Environmental context and resources	External factors
Social/professional role and identity	Reputation, professional relations, respect towards prescribers
Intentions, goals, optimism	Intentions, goals to change, believing that the change is something positive
Beliefs about capabilities	Doctors: Belief that one can stop the prescription of unnecessary antibiotics Patients: Belief that one can stop requesting antibiotics from doctors
Belief about consequences	Belief on outcomes if prescribers do not reduce prescription of broad-spectrum antibiotics / if patients do not stop demanding antibiotics
Reinforcement	Positive or negative feedback, follow-up for prescription of consumption of antibiotics
Emotions	Feelings that impact change positively or negatively

### Qualitative study data collection tools

The study relied on a mix of in-depth interviews and focus group discussions. Semi-structured question guides were developed for both based on the 14 domains of the TDF.

### Sampling of study subjects

The MOH purposively selected eight PHCCs for the pilot distributed geographically across the city to capture centres that served both low-income and middle-income patients and that had from 2–5 doctors on staff and an average of 660 patient visits per month.

All prescribers and patients who were available and interested to join the study during the data collection days were included in the sample. Data saturation was the principle for obtaining an adequate sample size. However, a minimum sample size for initial data collection was set at 10 in-depth interviews for prescribers and eight focus group discussions for patients, followed by three additional in-depth interviews until new themes were no longer produced [37].

### Data collection for the qualitative study

A field team of seven national and international public health professionals with previous experience in qualitative data collection conducted in-depth interviews with prescribers and focus group discussions with patients in the primary healthcare units. They received training to learn about the study and to gain more insight in to qualitative enquiry. The mixed group of data collectors was utilized to encourage different types of discussion to add to the richness of data. In-depth interviews were considered the most practical data collection method as the interviews were conducted during working hours in between patients or at the end of the working day. Focus group discussions were considered a suitable data collection method for patients who were recruited from the waiting areas of the primary healthcare units as they typically wait for some time for their turn to visit the doctor accordingly they have time “to waste”. Focus group discussions were not only considered a practical data collection method but also a highly acceptable approach; patients who were chatting with their friends in the waiting room often continued talking with the same familiar group of people in a private room during the focus group discussions which were often almost organic continuations of the waiting room chats with each other. Moreover, antibiotic consumption was not considered a sensitive topic that could not be discussed in a group setting. Patients were recruited from the waiting areas of the health unit by explaining the study purpose and, if interested, the patients were directed to a closed room where the focus group discussions took place. The focus group discussions were coordinated with the health facility nurse to ensure participants did not miss their appointments. A nurse also coordinated the recruitment of prescribers. Whenever a doctor had time for an interview, the nurse escorted the interviewer to a private room for the interview.

Data collection began by piloting and modifying question guides followed by conducting an average of 3–4 in-depth interviews or focus group discussions daily. The in-depth interviews lasted 25–40 min while focus group discussions were from 60 to 90 min. Informed written consent was obtained from all participants. All in-depth interviews and focus group discussions were conducted in Arabic, in a private space, and no identifiers were collected to ensure confidentiality. One field team member carried out the in-depth interviews and two conducted focus group discussions. Each day ended with a debriefing session where the teams shared key findings.

### Analysis of qualitative data

Data analysis began in the field with debriefing sessions that generated field notes used as a data source for triangulation purposes. Four members of the research team, who were also part of the data collection team, participated in the analysis. TDF served as a framework for analysis meaning that the narrative data was organized based on the domains [38]. The audio data was analysed using a methodology called rapid identification of themes from audio-recording that allows researchers to code and analyse qualitative data by transcribing only the parts that relate to the TDF drivers of behaviour [39].

The team discussed the TDF domains of the framework to reach to an understanding of how each domain was defined. Then, they listened to participant responses from audio recordings, considered their relevance to the TDF domains, transcribed and translated the relevant sections from Arabic to English directly from the audio recordings, and added them to a chart based on the TDF domains. Once the transcribed sections were added to the chart, they were reviewed by the lead investigator and re-discussed with the team to reach consensus. This was followed by the identification of codes and themes in each TDF domain based on the principles of thematic analysis that aimed to identify patterned meaning across datasets. The themes were derived inductively to condense raw text data into a brief, summary format that gives meaning to each TDF domain. The codes and themes were reviewed by other team members before the final interpretation of the data, which included developing a set of overarching themes for all relevant TDF domains and identifying all appropriate intervention functions to be discussed in the knowledge co-production workshop [39].

The study received ethical approval by the Ministry of Health in Sudan review board in January 2019 followed by the approval of the WHO Eastern Mediterranean Regional Office ethical board. All participants signed written consent forms. All interviews and focus group discussions were conducted in a private room and all interviews were anonymous.

### Workshop

A multidisciplinary group of over 70 Sudanese and international experts in public health, pharmacology, social sciences, and communication as well as primary healthcare physicians participated in a 3-day workshop in Sudan’s capital city of Khartoum. They included experts at the federal, state, and facility levels. The MOH project focal point invited the participants to join the workshop with the objective of developing a culturally acceptable and practical behaviour change intervention that can be implemented on a large scale. Participants were divided into six groups. Half of them worked with the prescriber-related data and the other half with patient-related data.

**Fig. 1:**
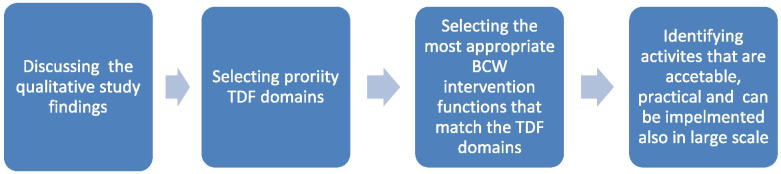
Workshop process

The workshop was based on three focus areas that built on one another. The workshop started with group discussions about the findings of the qualitative study to verify them and to identify priority TDF domains for both prescriber and patient interventions. The aim was to choose the most relevant domains for the context, which meant being of importance. Each group presented their prioritization of domains and their rationale. The discussions continued until a consensus was reached. Once workshop participants reached consensus, they moved on to selecting the most suitable BCW intervention functions that matched the selected TDF domains. Moderator of the session introduced BCW intervention function-initiated discussion about the most suitable intervention functions in the context of primary healthcare settings in Sudan. Moderators asked participants to think what intervention functions were practical and culturally acceptable and could be implemented in large scale. Once the workshop participants reached consensus on the intervention functions, they discussed activities that were practical, acceptable, and could be implemented on a large scale. Figure 1 illustrates the process. The discussions, which drew on expert knowledge and local understanding, carried on until consensus was reached. All workshop discussions were documented by note takers. The outcome of the workshop was consensus on TDF domains, intervention functions, and activities for the behaviour change pilot.

## Results

This section starts with the prescriber-related findings of the qualitative study and the knowledge co-production workshop followed by patient-related qualitative findings and workshop discussions.

### Prescribers

#### *Qualitative formative study*

##### Participant characteristics

The sample included 20 prescribers including general practitioners (50%), family medicine practitioners (44%) and dentists (6%). Most were females (69%) and from Gezira state (82%). Prescribers’ professional experience ranged from 1 to 24 years (median = 5.4 years).

##### Factors influencing antibiotic prescription practices

Seven of 14 TDF framework domains influencing prescription practices were identified: (1) knowledge, (2) skills, (3) social influences, (4) intention, (5) beliefs about consequences, (6) emotions, and (7) environmental factors.

##### Knowledge

Respondents explained that they do not have enough knowledge on the various classifications and types of antibiotics or the clinical indications. Many of them believed that their knowledge was outdated. Some respondents also highlighted that they rarely met representatives from pharmaceutical companies whom they considered potential sources of new information on antibiotics.

I do not think I know much about antibiotics or antibiotic prescription. It is not enough. (A male general practitioner)

Most respondents explained that antibiotic prescription-related decision making was influenced by various habits they developed over the course of their careers. For example, some explained prescribing antibiotics for all patients with symptoms of urinary tract infections or typhoid. Others described that they always prescribe broad-spectrum antibiotics to patients at risk of secondary bacterial infections.

Many respondents explained witnessing patients with antibiotic resistant infections in their practice but did not know the actual scope of the problem globally or nationally.

Yes, we see cases of resistant bacteria. We just change the antibiotic until it works. I don’t know if there is more resistance than before. (A male general practitioner)

##### Skills

Most respondents felt that they lacked the skills to convince patients who demand antibiotics that they are unnecessary. This was considered particularly challenging with patients who had predetermined the need for a specific type of antibiotic and those who had health insurance that provides free medications

I am not skilled enough to convince my patients. They come here with the antibiotic prescription in mind. It is difficult to make to accept that antibiotic is not needed. (A female general practitioner)

##### Social influences

Respondents’ social networks were often mixed with their professional networks meaning that patients were often also friends, neighbours or family members. Many prescribers pointed out that this made their refusal to prescribe unnecessary antibiotics difficult.

It is hard if I know the patients and I know his family. I am embarrassed to deny his requests. It is not easy. (A female general practitioner)

Whereas others noted the opposite saying that respect and trust among family and friends made antibiotic negotiations with them easier.

My family always consults with me, they trust me and I always give them my honest answer to everything. So I don’t have pressure to prescribe from that direction. (A female general practitioner)

Some respondents explained that patients also commonly pressured them emotionally to prescribe antibiotics by presenting themselves as underprivileged citizens for whom antibiotics were the only available resource.

Patients have their ways to make me feel guilty if I don’t prescribe them the kind of antibiotic they like to take. It is as if I took away the only thing they can have. (A female general practitioner)

Some respondents explained that their practices were guided by the practices of their patients. For example, patients are not accustomed to making follow-up visits so they prescribe antibiotics for potential bacterial infections.

I have patients who walk three hours to come to the facility, so I know that they won’t be coming back for a follow-up visit. I am accustomed to prescribing broad-spectrum antibiotics for them every time. (A female dentist)

Several respondents noted that they were influenced by patients’ antibiotic self-medication practices as many patients had a habit of taking antibiotics for long periods of time before coming to the health centres. Respondents noted that in those cases narrow spectrum antibiotics are no longer effective.

Our patients have often taken antibiotics for several weeks before they come here (health centre). For sure I need to prescribe strong antibiotics. Nothing else works. (A female general practitioner)

##### Intention

Most respondents had no intention to change their current practices. Many of them had not paid attention to their antibiotic prescription practices and accordingly they did not see any urgency to change them to be more aligned with the national guidelines

No, I don’t think this is what I will be doing (change current practices). In general, doctors are moving to stronger antibiotics, especially in private clinics. (A male general practitioner)

##### Beliefs about consequences

Some prescribers explained that they are observing an increasing number of patients with recurrent infections, a sign that stronger antibiotics and, in particular, broad spectrum antibiotics are needed unlike what the national guidelines recommend.

I can feel that we have more and more people with recurrent infections. This means that we cannot prescribe simple antibiotics any longer. (A male general practitioner)

##### Emotions

Many respondents also highlighted that the decision-making process was influenced by work exhaustion; when they were overwhelmed from work, they respond to the patients’ requests and prescribed unnecessary antibiotics without trying to reason with them. Respondents also clarified that work in the health facilities often included rush hours during which there was limited time and energy to discuss with patients and when prescribers easily gave into patient requests for antibiotics that were not necessary.

But when you are tired, you have no energy to debate with your patient. (A female general practitioner)

##### Environmental context and resources

Many respondents explained that the availability of antibiotics was rather limited in PHCC, and the availability of broad-spectrum antibiotics has increased over the years, which also encouraged their prescription practices.

Most of the time I need to consider what is available in stock not what I would like to prescribe. (A female general practitioner)

#### *Knowledge co-production workshop for prescriber intervention*

Of the seven TDF domains that emerged from the qualitative study, the workshop participants identified four that they considered priorities for the behaviour change strategy: (1) knowledge, (2) skills, (3) intention, and (4) social influences.

Knowledge was selected as increasing the knowledge of prescribers was seen essential. Antibiotics and AMR were not considered well covered in the medical curricula, and additional learning opportunities rarely addressed these issues, particularly in the rural healthcare setting. In addition, many physicians did not necessarily have access to smart phones or computers to seek information.

The workshop participants discussed the appropriateness of educational intervention, which was the BCW intervention function corresponding with knowledge. They pointed out that those education sessions would be acceptable to prescribers and they would be somewhat motivational. However, workshop participants concluded that educational sessions need to be short to be practical and to attract the participation of physicians.

Skills to address communication skills was seen as the second most relevant barrier as it was considered a persistent problem that consumed a significant amount of physicians’ energy on a daily basis. The workshop participants worked in small groups to discuss the appropriateness of training as an intervention function, which was the BCW intervention function corresponding with skills. They agreed that training was a viable approach to address physicians’ lack of communication and negotiation skills to convince patients not to take unnecessary antibiotics. Workshop participants concluded that physician training in Sudan does not include communication, therefore increasing the importance of this type of intervention function. Workshop participants believed that encouraging such training might be difficult among physicians as it is a non-medical topic. Accordingly, it was noted that the introduction of such an intervention requires careful planning, and it should be short and scheduled to best accommodate the physicians. Both education and training were considered interventions that can be also implemented on a large scale if needed as the MOH has training facilities and systems to cascade such interventions.

Limited intention to change current practices was selected as a priority domain as it was considered deeply embedded in the healthcare culture and in rural areas in particular where prescribers were less exposed to new ideas and trends than in cities. Workshop participants discussed the appropriateness of education and persuasion, as the BCW intervention functions that are linked with the TDF domain intention. They agreed that the best way to encourage intention to change behaviour was to use persuasion to promote ethical principles. Workshop participants explained that ethics as a topic was something that was perceived positively by the prescribers and something they all had learned in the past but which was forgotten amidst everyday routines.

Lastly, the workshop participants prioritized social influences as a priority domains as prescribers’ patients are commonly friends, neighbours or family members which influence their prescription practices. The workshop participants believed that there was a need to increase the self-confidence of the prescribers to prescribe based on what is medically correct, not based on what patients wished to receive. Modelling using prominent doctors as role models was selected as an intervention function. Modelling was considered highly context appropriate because the Sudanese health system is highly hierarchical and senior doctors are deeply respected and, accordingly, could be used to inspire change. The workshop participants highlighted that these role models should be local so the prescribers could relate to them and actually model this behaviour.

Workshop participants agreed that persuasion and modelling would be implemented via so-called peer visits over a period of 8 weeks to allow time for the prescribers to learn and process new concepts and model the new behaviours. Part of the visits would be conducted by selected local role models. They agreed that facility-based visits were not necessarily the most practical way to implement the intervention functions but face-to-face contact was seen as overriding the impracticalities of the approach as in the Sundanese culture face-to-face communication is highly appreciated. However, large-scale implementation would require further planning with the MOH. Workshop participants also agreed that using posters and video clips of role models would reinforce their involvement. Workshop participants decided to add a WhatsApp group as another communication channel to complement the facility-based visits. WhatsApp has become a common platform to communicate with friends and colleagues in Sudan; it is practical and can be implemented in large scale. The behaviour change framework for prescribers can be found in Table 2.

**Table 2:** Behaviour change framework for prescribers

TDF domain	COMB	Barrier	Objective	Intervention function
Knowledge	Capability	Lack of knowledge of antibiotics and AMR	Improve knowledge about the principles of antibiotic therapy, the epidemiology of AMR	Education
Skills	Capability	Lack of communication skills	Improve negotiation sills to answer patient demand	Training
Social influences	Opportunity	Patient views influence prescription practices	To enhance self-confidence by showcasing prominent doctors as role models for antibiotic prescription	Modelling
Intention	Motivation	No intention to change behaviour	Motivate change by promoting ethical principles and by giving examples of change	Persuasion

### Patients

#### Qualitative formative study

##### Participant characteristics

Eight focus group discussions were conducted with 94 patients. The majority were women (73.4%) originating from Gezira state (97.9%). Participants had completed various levels of education including university (32%), preparatory school (6.3%), secondary school (36.2%) or primary school (10.6%). Some respondents had no schooling (8.6%).

##### Factors influencing patient behaviour

Five domains of TDF were identified related to patient demand for antibiotics: (1) knowledge, (2) beliefs about consequences, (3) social influences, (4) social/professional role and identity, and (5) intention.

##### Knowledge

Respondents used antibiotics based on their knowledge about the need for antibiotics with specific symptoms or illness. Some respondents explained that antibiotics work with any type of health problems; others clarified that they used antibiotics whenever they had symptoms of a common cold such as runny nose or sore throat. Some respondents highlighted that they use antibiotics when they got malaria, body pain or general fatigue.

##### Beliefs about consequences

Respondents shared number of community perceptions and beliefs that were prominent. Most respondents highlighted that antibiotics were the fastest and the strongest type of medication, and the more expensive the better the antibiotic. Accordingly, imported antibiotics were believed to be better than local brands that were usually cheaper.

I always insist on the Jordanian ones; they are more expensive than the Sudanese ones. That’s why they are better. (Woman from Debagha village)

##### Social influences

Many respondents emphasized that people typically believe that one could not recover from infections without the use of antibiotics, which has made antibiotic use habitual for treating most health problems.

Respondents also cited some common community-based antibiotic practices such as keeping antibiotics at home in case someone gets sick and stocking up on antibiotics from the health unit before the holidays.

In addition, some respondents clarified that there were common preferences for the type of antibiotics among community members and usually the preferred type is used for any kind of health problems

In the community where I live, we commonly use Cipro for typhoid and Amoclan for any other problems. They work well. (A woman from El Mekky village)

Many respondents highlighted believing in their ability to diagnose their illnesses and to choose the type of antibiotic they required, especially for common infections. Likewise, they cited knowing what kind of antibiotic works best for them; not all antibiotics were believed to work for everyone.

I know what is effective for me, not the doctor. She does not know me, so it is difficult for her to know what works best. (A woman from Debagha village)

However, most respondents also mentioned the important role their families, friends, and neighbours played in advising them on the use of antibiotics. Usually elderly females in the household were the most knowledgeable. Mothers of young children frequently chatted with one another and learned about antibiotics through experience and word of mouth. Several respondents also clarified that villages had knowledgeable individuals who provided advice about antibiotics.

In our communities we have those whom we trust regarding health advice. They are part of our community and they know us. We share our illness histories with them. This is how life goes on in our communities. (A woman from Degbagha village)

##### Social/professional role and identity

Respondents frequently highlighted that many doctors prescribed the same antibiotic to all patients regardless of their health problem and they blamed physicians for not having enough information on antibiotics.

Some doctors prescribe the same antibiotic over and over again … they may not know much. (A woman from El Mekky village)

Pharmacists, on the other hand, were often seen as being able to diagnose patients and prescribe antibiotics. Respondents explained that they frequently consulted community pharmacists on antibiotic use.

If we talk about senior pharmacists, they are comparable to medical doctors. They are called doctors too.” (A man from Al Atra village)

##### Intention

Respondents had no intention to change their current practices of insisting on certain types of antibiotics.

I am not planning to stop asking for antibiotics. We need them. (A woman from Arkaweet village)

#### Knowledge co-production workshop for patient intervention

Of the five TDF domains that emerged from the qualitative study, the workshop participants identified two they considered priorities for the behaviour change strategy: (1) social influences and (2) intention.

Workshop participants agreed that (TDF domain) social influences were a priority because habitual antibiotic usage based on common community-based misconceptions was seen widely practised and that trust in antibiotics-related advice was placed in other community members rather than in prescribers. From the matching BCW intervention functions, the workshop participants selected enablement as it could be best used to change normative behaviours and trust building. Workshop participants also agreed that intention to change behaviour would be best addressed by an education intervention function that would focus on the implications of AMR.

The workshop participants continued to discuss activities that could be matched with enablement and education. They agreed that a face-to-face intervention was the most culturally appropriate. The participants proposed motivational interviewing or similar community outreach activities to tackle the habitual use of antibiotics, building trust towards prescribers, and education about the implications of AMR. However, workshop participants did not perceive it as practical and large-scale community outreach programmes were seen as difficult to implement as they are uncommon. Despite these difficulties, workshop participants believed that it was possible to find local resources such as community-based volunteers that can implement such community-based interventions. The behaviour change framework for patients can be found in Table 3.

**Table 3:** Behaviour change framework for patients

TDF domain	COMB	Barrier	Objective	Intervention function
Social influences	Opportunity	Antibiotic use habits are deeply rooted in community-based misconceptions Trust in community advice	Breaking normative behaviour and linked misconceptions Building trust between prescribers and patients	Enablement
Intention	Motivation	No intention to stop requesting antibiotics.	To discuss the implications of AMR	Education

## Discussion

To our knowledge, this is the first theory-based behaviour change intervention that applied the TDF and BCW to improve physicians’ antibiotic prescribing practices and to reduce patient demand for unnecessary antibiotics in primary healthcare settings in Sudan. This study contributes to the knowledge base by providing evidence to understand factors that are influencing antibiotic prescription and patient demand in low resource settings. This study adds to the growing body of evidence about the utility of TDF and BCW and provides suggestions for appropriate intervention functions on which to model future behaviour change interventions in similar settings.

The findings of the qualitative study have provided valuable information about the antibiotic prescribing and consumption culture in Sudan. The study found that these processes are complex and influenced by knowledge, social and cultural factors alike. The study identified eight overlapping TDF domains representing barriers to prescribers’ appropriate use of antibiotics, three of which were selected by the knowledge co-production workshop participants to be addressed in the behaviour change intervention: knowledge, skills, and intention to change behaviour.

The study also identified five TDF domains that affect patients’ demand for unnecessary antibiotics, two of which were included in the behaviour change strategy: social influences and intention. The study demonstrated the complexity of the factors that are linked with the behaviours and the importance of developing a multifactorial intervention as identified in several studies [40–42].

The study findings align with the growing body of research noting that although sufficient knowledge is likely to be a prerequisite for the appropriate prescription of antibiotics, increasing knowledge alone has little impact on changing antibiotic prescription practices unless all behavioural determinants and social norms influencing behaviours are addressed [18, 43, 44].

An important finding of the qualitative study was that both prescribers and patients lacked motivation to change their current practices. They did not think that their current antibiotic prescription or consumption practices required modification and, therefore, had no intention to change. This finding is somewhat contradictory to recent studies in other countries where healthcare workers admitted the need to make a change and in some cases they had even made plans to modify their practices [45, 46]. The efforts to identify more effective methods of changing intentions should also include the promotion of intention stability and sustainability [47].

Lack of communication skills to answer patient demand was a prescriber-related driver of unnecessary antibiotics prescription practices. Convincing patients during short consultations was considered challenging. A recent study in Tanzania concluded likewise that an emphasis on communication skills is crucial in helping physicians deal with patients who demand antibiotics [48]. Moreover, a review of the effectiveness of physician-targeted interventions to improve antibiotic use found that communication skills training had one of the largest effects on behaviour change [49].

The findings of the qualitative study also indicated that patients trusted their social networks more than physicians regarding antibiotics, which highlights the importance of social factors and trust building components. Trust among family and friends with regard to health-related matters has been identified in many cultural settings [50, 51].

Knowledge co-production also enriched the discussions [33], which culminated in the identification of four BCW intervention functions for prescribers to address the selected TDF behavioural domain: education, training, modelling, and persuasion and as well as two BCW intervention functions for patients: education and enablement. The discussions also identified appropriate activities to match with the intervention functions. Knowledge co-creation also ensured that the planning took into consideration local practicalities of the interventions. Previous studies highlight that consideration of local issues, matters, and community structures are of utmost importance to the success of the intervention [27].

Previous studies in the region indicate that educational interventions alone have limited impact to change behaviours [48, 52, 53], but educational components are included in many successful multifaceted interventions [4, 12, 54, 55]. Enablement interventions are specific for intervening on factors that block the behaviour change [20]. In this project, it included breaking normative behaviour and linked misconceptions and building trust between prescribers and patients.

Successful norm changes are often combined with policy changes. Therefore, a policy analysis could be a beneficial methodology to understand and intervene in the policy environment [56, 57]. For example, in Sudan, the national health insurance policy, which offers free medical services and a 25% patient co-payment system for medications, is likely encouraging habitual antibiotic use in Sudan which must be taken into consideration when aiming to change normative behaviours. Management trust building interventions that have worked in other countries should be considered [57]. Modelling interventions that use role models as change agents usually serve three distinct functions in which they influence goals and motivation: acting as behavioural models, representing what is possible, and being inspirational [58]. The selection of role model doctors that are expected to act as facilitators should be based on these factors.

The study had some limitations. The intervention focused only on behavioural factors although environmental factors such limited availability of narrow spectrum antibiotics was identified as a driver of unnecessary broad-spectrum antibiotics prescription practices. Addressing such structural changes was outside the scope of the project. Interviews with the prescribers may have been influenced with social desirability bias. The interviewers aimed to minimize the bias by helping the interviewees feel at easy by ensuring that their wording was encouraging and nonjudgmental. As a pilot, the scope of study was limited. To enhance generalizability, future studies should consider including prescribers from secondary and tertiary care levels and expanding covering to more geographic locations.

Based on the results of this study, a detailed behaviour change intervention strategy will be forthcoming and evaluated in Sudan to promote antibiotic prescription practices in primary healthcare settings in Sudan

## Conclusions

This study provided detailed insights into behavioural factors that influence the prescription and use of antibiotics among prescribers and patients, respectively, in primary healthcare settings in Sudan. The incorporation of behavioural theories—TDF and BCW—supported the identification of key factors that are integral to understanding antibiotic use in primary healthcare settings in Sudan: antibiotic use habits, social influences and lack of intention to change current practices as well as the importance of communication skills to convince patients not to use antibiotics unnecessarily.

## Data Availability

The datasets generated during and the current studies are not publicly available but are available from the corresponding author upon reasonable request.
